# Modern Views as to the Surgical Treatment of Fibro-Myomata of the Uterus

**Published:** 1897-07-10

**Authors:** Fred. Bowreman Jessett

**Affiliations:** Surgeon to the Cancer Hospital, Brompton


### MODERN VIEWS AS TO THE SURGICAL TREATMENT OF FIBRO-MYOMATA OF THE UTERUS.

By Fred. Bowreman Jessett, F.R.C.S., Surgeon to the Cancer Hospital, Brompton.

In no branch of surgery has there been such marked advances made as in the surgical treatment of abdominal tumours, and perhaps among these tumours the treatment of fibro-myomata of the uterus has attracted the most attention. The space at my disposal will not allow of my saying so much as I could wish upon the subject, and I shall limit my remarks purely to the advances made in the treatment of these tumours by abdominal hysterectomy.

The late Dr. Clay (Manchester) was the first surgeon who removed a myomata uterus: the first case by accident in 1843, and again in 1844, the tumours being mistaken for ovarium; both patients died. The operation was not attempted again until 1863, when Dr. Clay did his first deliberate hysterectomy. This case made a good recovery.

In the same year Kœberlé performed the second hysterectomy in Europe, and to him must be credited the discovery of the extra-peritoneal method of treatment by the "serre-nœad." This method of operating is that which has been adopted in this country until quite a recent date, the principle suggested by Kœberlé being maintained, although the details of the operation have been very much improved upon by Pean, Keith, Bantock, and Thornton. By the work of these surgeons, and strict attention to the *technique*, the mortality has been reduced approximately to that of ovariotomy.

This extra-peritoneal method of treating the stump was, however, looked upon by many surgeons as far from coming up to an ideal operation. The patients suffered considerable pain from traction on the stump, Convalescence was slow, the parieties were weakened, and ventral hernia was not an uncommon occurrence. To avoid these disadvantages Schroeder in 1882 published a method, in which he planned out a systematic operation for the extra-peritoneal method of operating on the myomatous uterus, and there can be but little doubt, had he lived longer, to him would have been the credit of perfecting the "technique" of this operation. It is true his results were poor, but that was due chiefly to asepsis.

Schroeder thought it necessary to use the elastic ligature in his operation, and this necessitated his amputating the uterus at a high level, hence he ligatured the uterine arteries after they had given off the vaginal and cervical branches, instead of ligaturing the trunk of the artery; thus, on removal of the elastic ligature, smart hæmorrhage occurred in the stump, necessitating the application of numerous ligatures. Finally, the amputation of the uterus at so high a level left a large, raw surface, which, it is true, he covered with peritoneum, but he did not recognise the necessity of covering the wounds in the broad ligaments with peritoneum, and by so doing making the whole field of operation retro-peritoneal.

The results of the operation thus carried out were so discouraging that it fell into disuse. Dr. Bardenheuer about this time published a work giving his experience of total hysterectomy for fibroid tissues, and was the first surgeon in Europe to practise this form of operation which was afterwards practised by Martin (Berlin), Fritsch, and others in Germany. Neither of these operations, however, found favour in this country, and it was not until Dr. Milton, of Cairo, published a series of cases in the *Lancet* in November, 1890, of the extra-peritoneal treatment of the stump, that any real progress was made. This paper, however, attracted much attention, and I in 1891 operated successfully on a similar case. Dr. Heywood Smith about the same time also performed successfully Milton's operation, and read a paper upon the subject advocating this plan, which he preferred to call sub-peritoneal hysterectomy. In this paper he points out very clearly the advantages of the retro-peritoneal treatment of the stump. His cases were not, however, altogether satisfactory, as in each of the three cases he relates suppuration or hæmatocele occurred. I think this was to a very great extent due to his use of the elastic ligature necessitating the ligaturing of uterine arteries too high up, and thus, on removal of the elastic ligature, considerable oozing or bleeding in the stump occurred.

Other surgeons who have adopted this operation were in the habit of cauterising the cervical canal leading into the vagina, for fear of infection from this source. The researches of Döderlein, which show that the cervical canal, when not interfered with, does not contain septic organisms, clearly, I consider, show that this treatment of the cervical canal, far from doing good, may be the very means of causing septic mischief.

Dr. B. F. Baer, of Philadelphia, first performed the operation in 1891, and published the results of his work in 1892, which were splendid. In his paper he lays great stress on controlling all hæmorrhage; the non-constriction of the cervical tissues, so that there shall be no fear of suppuration; and, lastly, the non-disturbance of the cervical canal, so that sepsis from the vagina may be prevented.

At the same period having performed the sub-peritoneal hysterectomy in a few cases myself, although I recognised the great advances of this operation upon the supra-vaginal (extra peritoneal) by Kœberlé method, yet I felt it was not an ideal operation, and I was convinced that removal of the whole organ was the more scientific procedure, as by it perfect drainage could be ensured through the vagina, and the patient should make as good a recovery, if care was taken to thoroughly disinfect the vagina, as after vaginal hysterectomy for carcinoma of the uterus; and, as in this class of cases I had operated upon a large number of cases with a mortality of about some 8 per cent., I determined to practice total abdominal hysterectomy on the first favourable case that presented itself. In July, 1893, I performed my first operation of total removal (pan-hysterectomy) successfully; this was followed by others, all the patients recovering just as after ovariotomy. I have now performed this operation on over twenty cases, with a mortality of about 8 per cent. There are cases undoubtedly in which the sub-peritoneal method is preferable, but in a large majority of cases I am convinced that total removal is the better method. The cases that cannot be treated by either of these methods must be few indeed, and I do not think the day is far distant when Kœberlé's serre-nœad will be delegated to the museum as an instrument which has done excellent work, but one which the progress of surgery has banished.

I consider that many cases of patients suffering from myomata of the uterus are lost, or allowed to linger on in miserable existence, because they are not operated on early enough. The tumours are allowed to attain enormous dimensions, when interference is out of the question; or adhesions are formed which make the operation much more difficult and hazardous.

The chief points to consider to ensure success are: 1. Thorough asepsis of the vagina. This is best attained by frequent douching with hyd. perch. solution, and the insertion of a tampon saturated in the same solution into the vagina. 2. The perfect disinfection of abdominal parieties. 3. At the time of the operation placing the patient in Trendelenburg's position, so as to insure a good view of the pelvic floor. 4. The ligaturing of the ovarian and uterine arteries securely at their trunks. 5. In cases of complete removal to see that the peritoneal flaps are everted into the vagina, and that the remainder of wound in the broad ligament is carefully stitched over with peritoneum. In cases of sub-peritoneal hysterectomy to see that the stump and wound in the broad ligament are covered by peritoneum carefully stitched over. 6. The avoidance of the use of the elastic ligature during the operation, to prevent any bruising or injury to the parts. 7. The careful packing of the vagina with iodoform gauze to ensure free drainage and to prevent any fear of the peritoneum being affected by any septic organism from without.

In this short review of the progress of the surgical treatment of fibro-myomata of the uterus I have only been able to allude to some few of the many surgeons who have done much to advance this department of surgery, but had I once launched out and attempted to describe the different methods of operating adopted by different surgeons this paper must have far exceeded the limits to which I am entitled.

